# Supplementary material for: Triamterene Functions as an Effective Nonsense Suppression Agent for MPS I-H (Hurler Syndrome)
Source: Int J Mol Sci. 2023 Feb 24;24(5):4521. doi: 10.3390/ijms24054521 (PMC10003437; doi:10.3390/ijms24054521)
Supplement: Supplementary file 1 [file ijms-24-04521-s001.zip › ijms-2230529-supplementary.pdf]

Supplementary Table S1: List of top hits identified using a NanoLuc dual readthrough/NMD reporter in the presence of low dose G418.

Compound	Function	Drug Conc.	Drug Alone Fold-Increase (rel. to basal)	+G418 Fold-Increase (rel. to G418 alone)
Imantinib	Brc-Abl inhibitor; anti-cancer	60 µM	5	59
Triamterene	ENaC inhibitor; diuretic for hypertension & edema	60 µM	13	38
AB00993554	SR compound	30 µg/mL	7	32
Doxazocin	α_1-sensitive alpha blocker; hypertension & urinary retention	60 µM	4	31
GS-6201	adenosine A_{2B} antagonist; anti-inflammatory	60 µM	10	31
AB00990535	SR compound	30 µg/mL	12	26
Verteporfin	photosensitizer to eliminate abnormal blood vessels in the eye	60 µM	4	22
AB00989308	SR compound	30 µg/mL	6	22
AB00993402	SR compound	30 µg/mL	10	20
AB00989525	SR compound	30 µg/mL	6	19
Tandutinib	FLT2 inhibitor; anti-cancer	60 µM	4	19
AB00990301	SR compound	30 µg/mL	7	18
Tadalafil	PDE5 inhibitor; hypertension; erectile dysfunction	60 µM	2	18
Naloxonazine	µ-opioid receptor antagonist	60 µM	3	18
Elacridar	ABC transporter inhibitor; tumor multi-drug Resistance	60 µM	5	17
Quinacrine	anti-malarial	60 µM	25	15
Vorinostat	histone deacetylase inhibitor; anti-cancer	60 µM	37	12
G418 alone	readthrough control	100 µg/mL	12	1

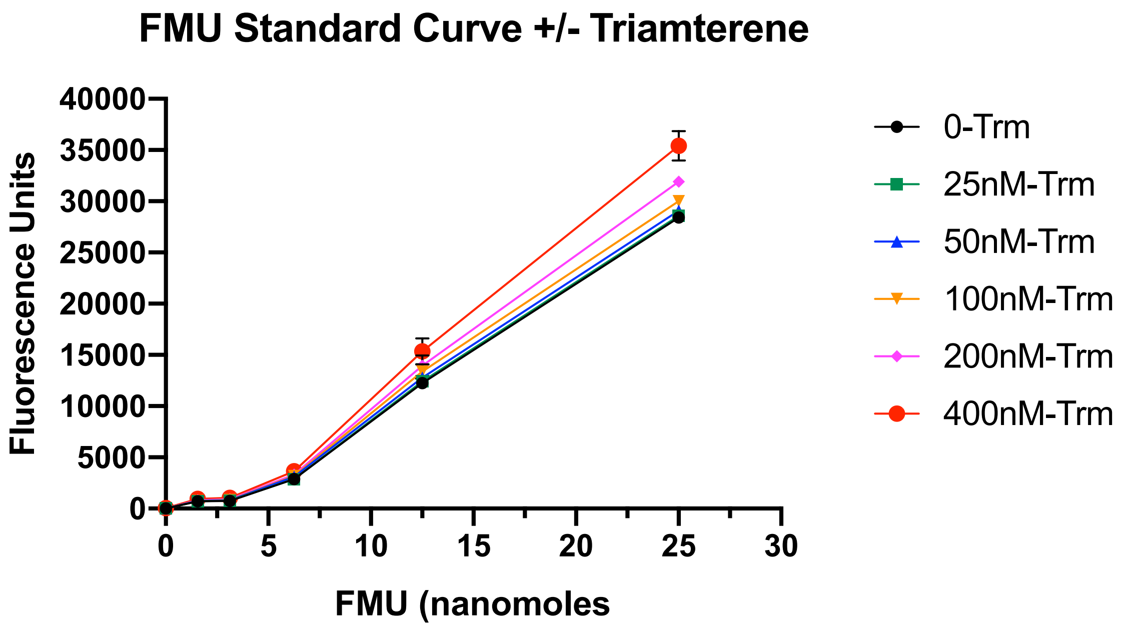

Supplementary Figure S1: FMU standard curve +/- triamterene. Standard curves were generated to calculate **α**-L-iduoridase activity by measuring the fluorescence units generated using free acid 4-methyl-umbelliferone (nanomoles) in the presence of vehicle only (0-Trm) or triamterene ranging from 25-400 nM. Each data point is the mean +/- SD of three replicates.

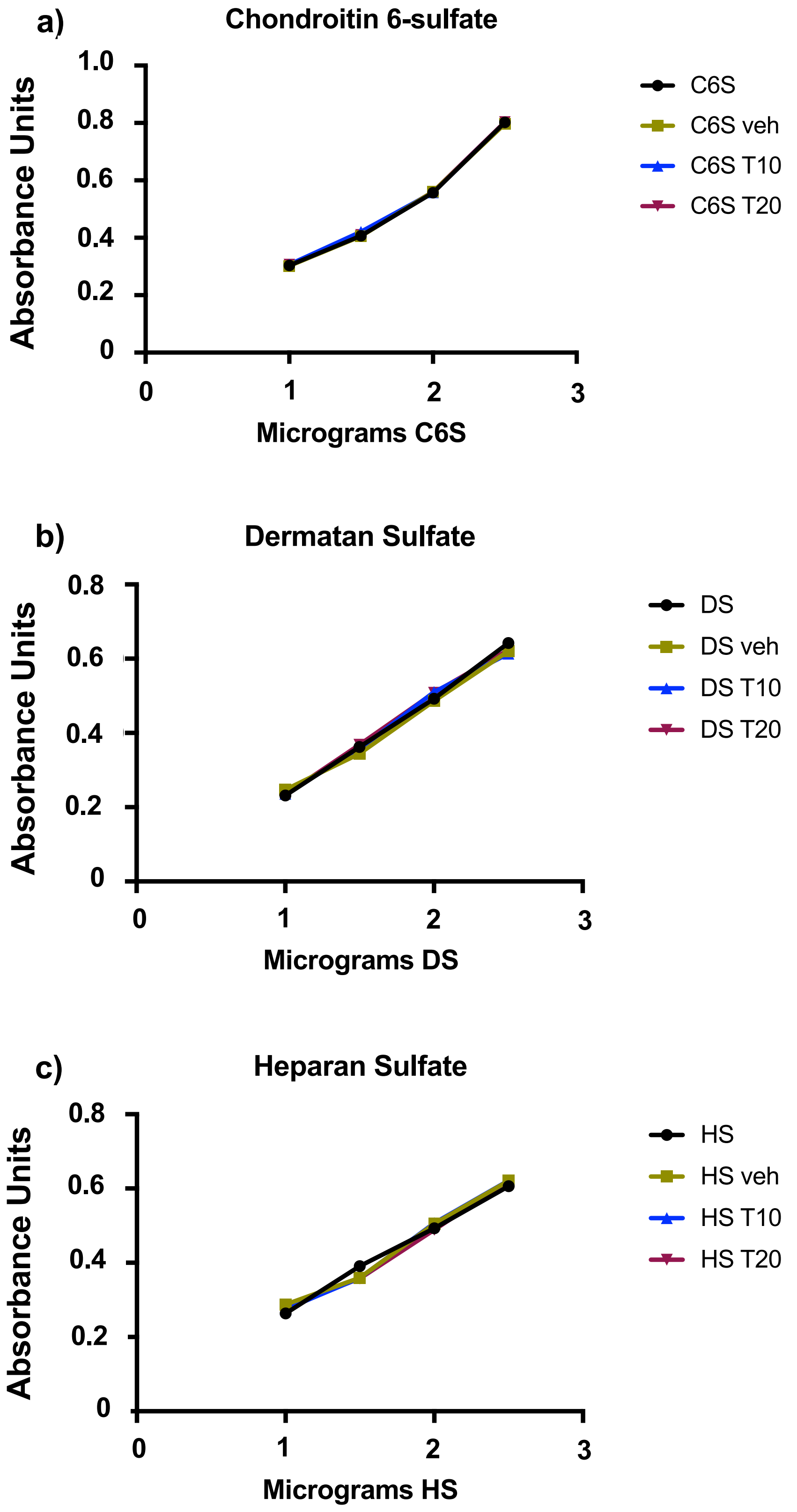

Supplementary Figure S2: Triamterene does not interfere with the GAG dye-binding assay. Standard curves were generated using the Blyscan GAG dye-binding assay with (**a**) chondroitin 6-sulfate (C6S), (**b**) dermatan sulfate (DS), or (**c**) heparan sulfate (HS) in the absence and presence of vehicle, T10 = 10μM triamterene, or T20= 20μM triamterene. Each data point is the mean +/- SD of three replicates.

a)

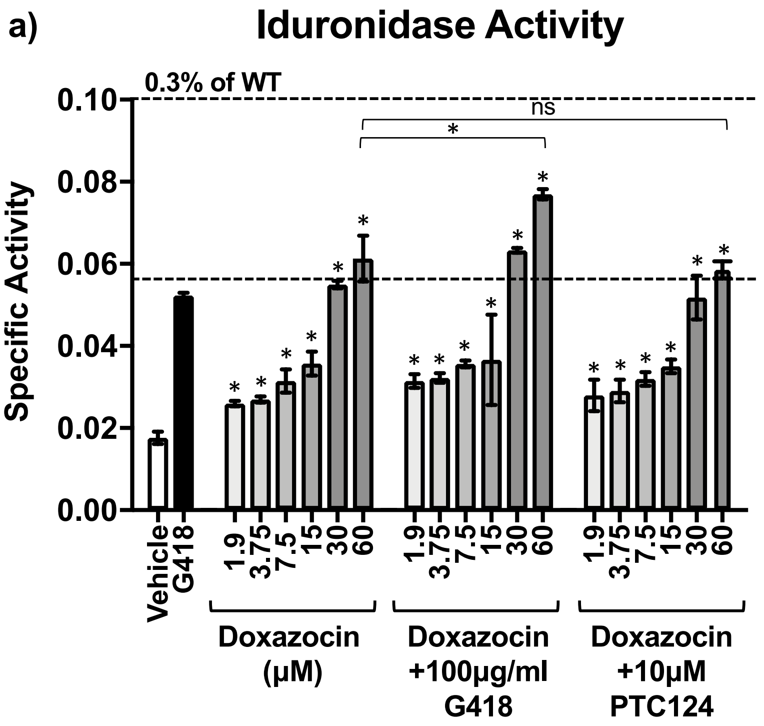

b)

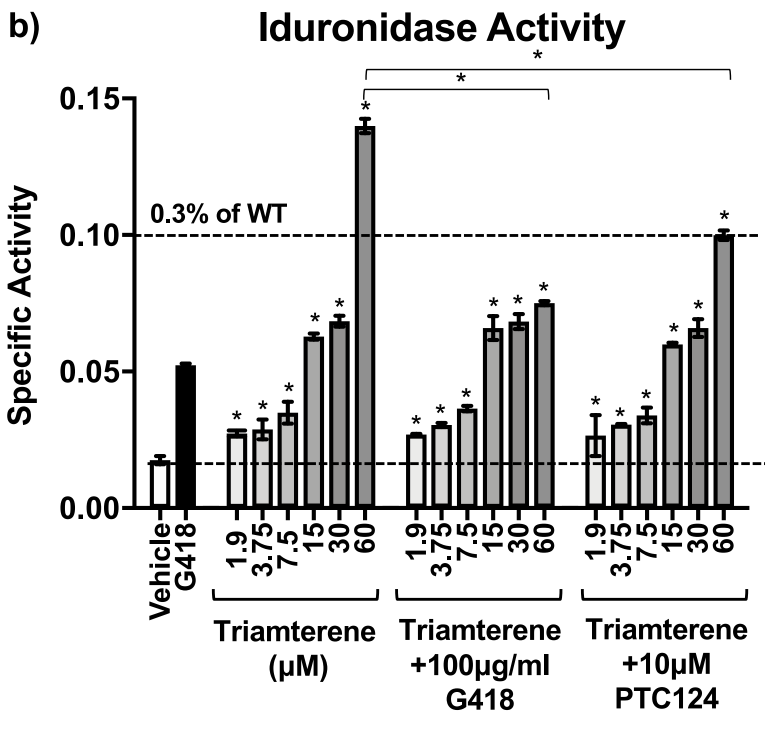

Supplementary Figure S3: Triamterene does not produce synergistic increases in α-L-iduronidase activity when combined with G418 or PTC124 in *Idua*-W402X MEFs. Each column represents the mean +/- SD of two independent experiments (n= 6-8). In panels a & b, the dashed line represents 0.3% of WT α-L-iduronidase activity. * above each column indicates $p<0.05$ when comparing treated samples with the vehicle and * above a bracket indicates $p<0.5$ when comparing the 60uM treatment alone when compared to 60uM combined with other readthrough compounds; ns = not significant ($p>0.05$).

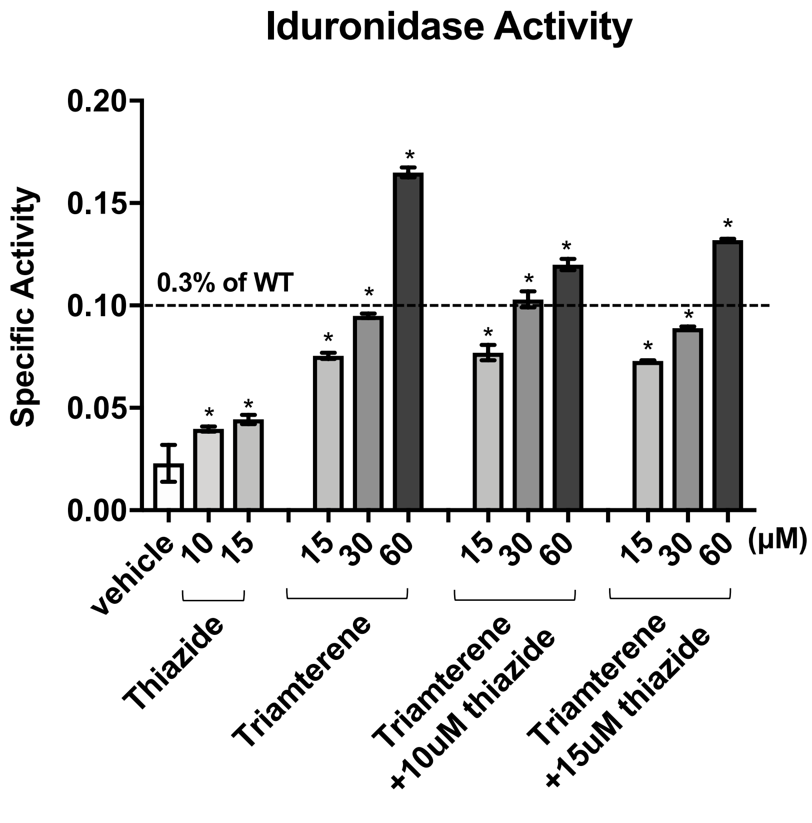

Supplementary Figure S4: Thiazide, a diuretic normally administered clinically with triamterene, does not interfere with the ability of triamterene to suppress the *Idua*-W402X mutation in MEFs. Each column represents the mean +/- SD of two independent experiments (n= 6-8). The dashed line represents 0.3% of WT α-L-iduronidase activity. * indicates $p<0.05$ when comparing treated cells to vehicle alone.

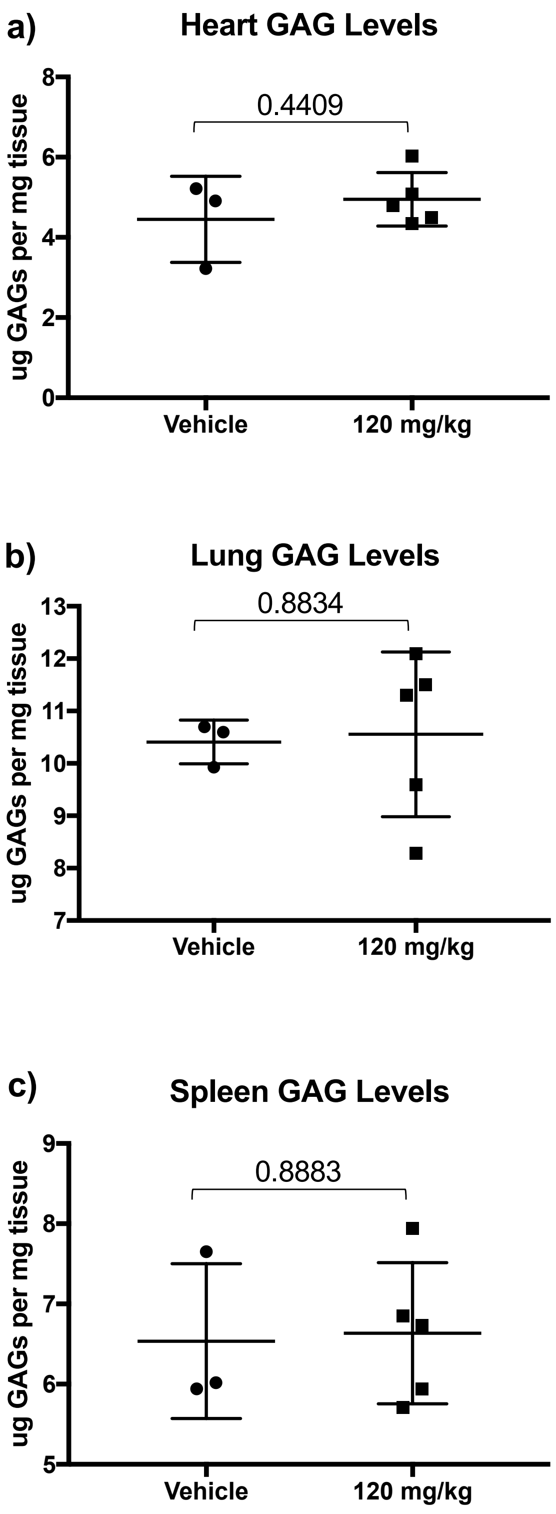

Supplementary Figure S5: Triamterene does not reduce GAG levels in *Idua*-KO mice. 8-week-old *Idua*-KO mice were administered triamterene once daily via oral gavage for a total of 2 weeks (14 doses). 24 hours after the final dosing, mouse tissues were collected upon sacrifice. Sulfated GAG levels in defatted, dried tissue homogenates were quantified using a GAG dye-binding assay [12]. GAG levels were quantified in the following mouse tissues: (**a**) heart; (**b**) lung; (**c**) spleen. Each point represents the average GAG value obtained from an individual mouse. Exact p values were calculated using a two-tailed t-test.

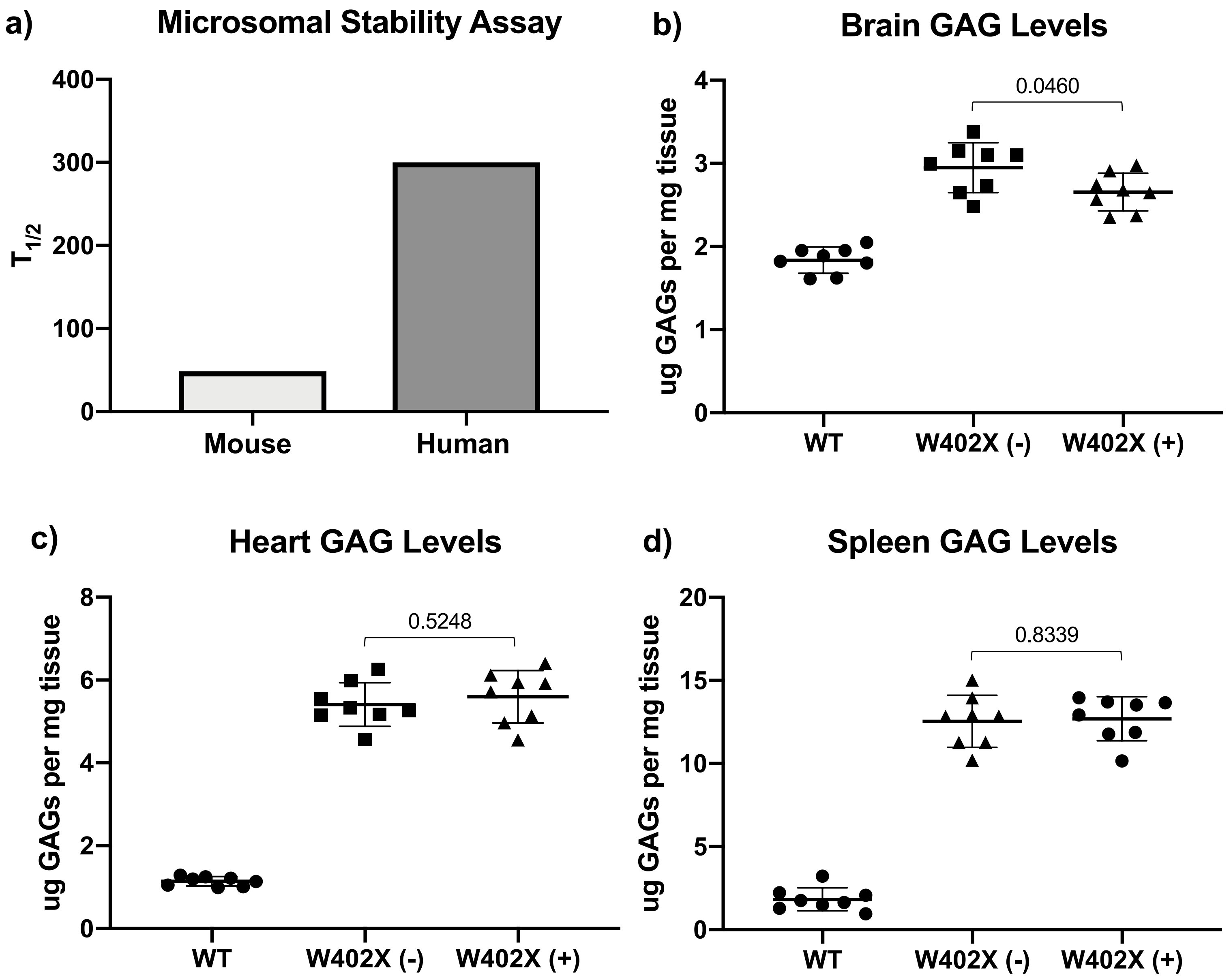

Supplementary Figure S6: Extensive triamterene metabolism in mice inactivates its ability to induce readthrough. (**a**) Microsomal stability assay comparing triamterene metabolism in human versus mouse microsomes; expressed as the time (minutes) at which half of the parental compound remains. Each column presents the mean of two replicate experiments. GAG levels in the (**b**) brain, (**c**) heart, and (**d**) spleen of *Idua*-W402X mice after 26-weeks of triamterene treatment (120 mg/kg once daily via oral gavage). Each point represents the average GAG value obtained from an individual mouse, with 8 mice included in each cohort. Exact p values were calculated using an unpaired, two-tailed t-test; the cohorts compared are indicated by the brackets. Note that $p<0.0001$ when comparing all WT and W402X cohorts.
